# High-Accuracy Detection of Odor Presence from Olfactory Bulb Local Field Potentials via Deep Neural Networks

**DOI:** 10.3390/s26030951

**Published:** 2026-02-02

**Authors:** Matin Hassanloo, Ali Zareh, Mehmet Kemal Özdemir

**Affiliations:** 1 Department of Computer Engineering, Istanbul Medipol University, Kavacık Campus, Istanbul 34810, Turkey; ali.zareh@std.medipol.edu.tr; 2 Department of Artificial Intelligence Engineering, Istanbul Medipol University, Kavacık Campus, Istanbul 34810, Turkey; mkozdemir@medipol.edu.tr; 3 Department of Computer Engineering, Ankara Medipol University, Ankara 06050, Turkey

**Keywords:** olfactory neural signals, deep neural networks, odor detection, extracellular recordings, time–frequency analysis, local field potentials, brain–computer interfaces

## Abstract

Odor detection underpins food safety, environmental monitoring, medical diagnostics, and many more fields. Current artificial sensors developed for odor detection struggle with complex mixtures, while non-invasive recordings lack reliable single-trial fidelity. To develop a general system for odor detection, in this study we present preliminary work where we test two hypotheses: (i) that spectral features of local field potentials (LFPs) are sufficient for robust single-trial odor detection and (ii) that signals from the olfactory bulb alone are adequate. To test these hypotheses, we propose an ensemble of complementary one-dimensional convolutional networks (ResCNN and AttentionCNN) that decodes the presence of odor from multichannel olfactory bulb LFPs. Tested on 2349 trials from seven awake mice, our final ensemble model supports both hypotheses, achieving a mean accuracy of 86.2%, an F1-score of 85.3%, and an AUC of 0.942, substantially outperforming previous benchmarks. The t-SNE visualization confirms that our framework captures biologically significant signatures. These findings establish the feasibility of robust single-trial detection of odor presence from extracellular LFPs and demonstrate the potential of deep learning models to provide deeper understanding of olfactory representations.

## 1. Introduction

The development of advanced sensor technologies for the reliable detection of odorants is a critical challenge in different fields ranging from environmental safety to medical diagnostics [1,2,3,4,5]. Moving beyond the limitations of conventional electronic noses (e-noses), the field of neural sensing aims to emulate the olfactory system, using its rapid and complex processing to create high-performance brain–computer interfaces (BCIs) [6,7,8,9,10,11]. Central to this approach is the decoding of neural signals, and among these, the LFP offers a uniquely powerful data source. Unlike the sparse firing patterns of individual neurons, the LFP represents the synchronized synaptic activity of thousands of neurons, offering a robust signal for single-trial classification [12,13,14].

LFP recordings overcome the limitations of non-invasive methods in two critical aspects. First, direct LFP recordings offer superior spatial resolution, eliminating contamination from non-neural sources [13]. Second, these recordings provide a high signal bandwidth that allows analysis of high-frequency oscillations known to be important for olfactory processing [15]. By capturing this clean and full spectrum of neural activity, LFP recordings offer a powerful and decodable feature source for the specific task of binary odor detection, potentially overcoming the limitations of traditional sensors.

Previous studies on the decoding of odor presence from neural signals have generally focused on non-invasive methods, which suffer from a critical loss of high-fidelity spectral information [16,17,18]. Scalp electroencephalography (EEG), for instance, struggles with a low signal-to-noise ratio (SNR) due to the deep cortical source of olfactory signals [19,20]. Consequently, achieving successful classification with these non-invasive signals requires methods that are impractical for real-world applications. For example, many studies rely on averaging responses across dozens of trials, making real-time detection impossible [21,22]. The challenge of non-invasive decoding is illustrated in a recent study by Rajabi et al., who targeted olfactory bulb (OB) activity non-invasively with an electrobulbogram (EBG) and a 1D CNN. Their model achieved an Area Under the Curve (AUC) of 0.58, indicating a performance close to the binary chance level for single-trial classification [23].

We introduce an ensemble of complementary convolutional neural networks, combining an AttentionCNN and a ResCNN, to determine odor presence from 32-channel extracellular LFP recordings. We propose that using high-fidelity direct LFP recordings instead of low-fidelity non-invasive signals will help close the existing performance gap. Based on the proposed ensemble model, we propose two central hypotheses. First, we hypothesize that the spectro-temporal features within single-trial LFPs provide sufficient information to robustly and accurately classify odor-presence versus odor-absence conditions. Second, we hypothesize that neural activity from the olfactory bulb alone is sufficient for the odor presence detection task, without requiring signals from downstream processing areas like the piriform cortex (PCx). By developing an ensemble of deep convolutional neural networks [24,25], we demonstrate a significant leap in performance, thereby establishing the feasibility of using LFP recordings as a foundation for odor sensing.

## 2. Materials and Methods

**Figure 1:**
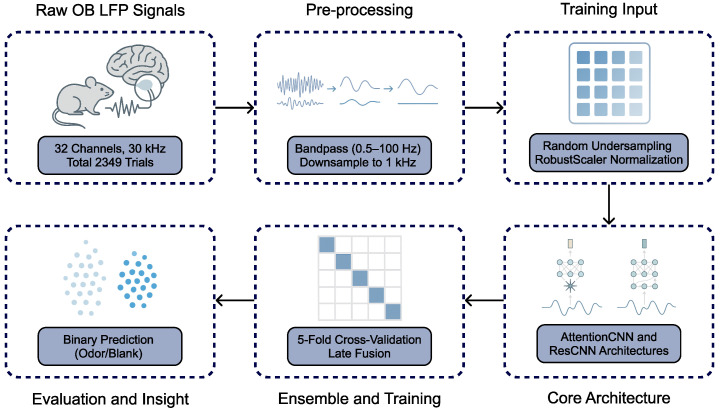
Workflow of the proposed model for odor presence detection from OB LFPs. Raw recordings undergo bandpass filtering, downsampling, and normalization before training complementary CNNs. Ensemble fusion enables binary odor classification and performance evaluation.

In this section, we detail the pre-processing steps applied to LFP signals, introduce two core architectures (AttentionCNN and ResCNN), and explain the procedures for training and ensembling these models, along with the evaluation metrics. Figure 1 provides an overview of our methodological pipeline.

### 2.1. Data Source and Experimental Context

Our analysis uses the *pcx-1* dataset [26,27] prepared by Bolding and Franks (2018), which is publicly available on CRCNS.org (https://doi.org/10.6080/K00C4SZB). It contains simultaneous extracellular recordings of mouse OB and PCx, acquired with 32-channel NeuroNexus Poly3 silicon probes (NeuroNexus, Ann Arbor, MI, USA) at a sampling rate of 30 kHz, together with respiration traces sampled at 2 kHz. Although both OB and PCx signals are provided, in this study we analyze only the OB recordings.

The dataset comprises two complementary recording sets. The main dataset contains 2349 trials from seven awake head-fixed mice, with odor stimuli (ethyl butyrate, isoamyl acetate, 2-hexanone, hexanal, ethyl acetate, and ethyl tiglate), each delivered at 0.3% *v*/*v* concentration; mineral oil served as the baseline control (*n* = 336 trials). A supplementary concentration dataset contains 2600 trials examining ethyl butyrate across four concentrations (0.03%, 0.1%, 0.3%, and 1.0% *v*/*v*; *n* = 200 trials per concentration) with matched mineral oil controls (*n* = 200 trials), to enable a systematic analysis of concentration-dependent detection thresholds. All analyses were implemented in Python (version 3.12.12) using NumPy (version 2.0.2) for numerical computations.

### 2.2. Pre-Processing

#### 2.2.1. Dataset Preparation and Class Balancing

A systematic pre-processing pipeline was implemented to prepare the raw LFP signals for analysis. The ‘Odor-Presence’ class was established from all active odorant trials (*n* = 2013) while the Mineral Oil controls (*n* = 336) defined the ‘Odor-Absence’ class. This grouping resulted in a significant 6:1 data imbalance. To mitigate the risk of classification bias, we applied random under-sampling (without replacement) to the ‘Odor-Presence’ group, selecting 336 trials to match the minority class. This procedure yielded a final balanced dataset composed of 672 trials for the binary odor detection task.

#### 2.2.2. Signal Filtering and Downsampling

Every 32 recording channels were subjected to a fifth-order Butterworth bandpass filter (SciPy version 1.16.3) with cut-off points at 0.5 Hz and 100 Hz. These cut-off points were chosen to allow odor-relevant frequencies in the delta to gamma range to be preserved and to eliminate baseline drift and high-frequency artifacts [14,28,29]. After filtering, the data were downsampled at a rate of 30 to 1 kHz, resulting in 2000 data samples per 2-s trial with no aliasing artifacts (since the cut-off is well below the Nyquist frequency at 500 Hz post-downsampling).

#### 2.2.3. Spectral Feature Extraction and Normalization

Following the pre-processing, spectral features were extracted and normalized. First, power spectral densities (PSDs) were computed for each channel using Welch’s method with a 256-point Hann window and 50.0% overlap [30]. The resulting spectra were then normalized using a RobustScaler (scikit-learn version 1.6.1) by subtracting the median and dividing it by the inter-quartile range to reduce the impact of spectral outliers [31].

### 2.3. Network Architecture

**AttentionCNN:** AttentionCNN is a well-known architecture for processing complex feature sets. Given the richness and variability of odor-evoked spectral activity, we chose this model to focus on the most discriminative temporal patterns. Our implementation begins with a stand-alone max-pooling layer (stride 2), which is followed by two identical convolutional blocks. Each block consists of the sequence [Conv1D → BatchNorm → ReLU → MaxPool (stride 4)], and these operations collectively expand the output to 128 channels. We then apply three parallel Conv1D branches (kernel sizes 1, 3, 5; 64 filters each), concatenate to form 192 channels, and then recalibrate via a squeeze-and-excitation channel attention module [32], followed by spatial attention.

For a given feature map $U\in R^{C\times T}$, the squeeze-and-excitation operation performs the following:

(1) $$\begin{array}{cc} z_{c} & =\frac{1}{T}\sum_{t=1}^{T}u_{c}\left(t\right)\;(\mathrm{global}\;\mathrm{average}\;\mathrm{pooling}), \end{array}$$

(2) $$\begin{array}{cc} s & =\sigma (W_{2}\delta \left(W_{1}z\right))\;(\mathrm{excitation}), \end{array}$$

(3) $$\begin{array}{cc} {\tilde{U}}_{c} & =s_{c}\cdot U_{c}\;(\mathrm{recalibration}), \end{array}$$

where σ is the sigmoid function, δ is ReLU, and $W_{1}\in R^{C/r\times C}$, $W_{2}\in R^{C\times C/r}$ with reduction ratio r=16.

A global average pooling layer reduces the temporal dimension, producing a 192-dimensional vector that passes through dropout (*p* = 0.3), a 256-unit ReLU fully connected layer, dropout (*p* = 0.5), and a final linear classifier for binary odor detection.

**ResCNN:** ResCNN builds on residual blocks to enable very deep networks, improving the gradient flow and feature reuse. This makes it ideal for capturing hierarchical temporal features in LFP spectra. Our ResCNN adaptation starts with max-pooling (stride 2), a Conv1D layer (kernel 7, stride 2) with BatchNorm and ReLU, and another max-pool (stride 4). Three residual blocks (64 channels each) then refine the features, followed by a Conv1D (kernel 3, stride 1 → BatchNorm → ReLU) to expand to 128 channels. After a final max-pool (stride 2) and two more 128-channel residual blocks, global average pooling condenses the temporal dimension. A dropout layer (*p* = 0.4) precedes the final linear classifier, producing robust single-trial odor-presence decisions.

### 2.4. Rationale for Architecture Selection

To justify our architecture choices and ensure reproducibility, we compared eight CNN variants on our dataset: (1) Vanilla CNN (3-layer baseline), (2) Deep CNN (6 layers without skip connections), (3) Dilated CNN (dilated convolutions), (4) Wide CNN (increased channel width), (5) Shallow CNN (2 layers), (6) AttentionCNN (our proposed architecture with CBAM attention), (7) ResCNN (our proposed architecture with squeeze-and-excitation residual blocks), and (8) Ensemble (combination of AttentionCNN and ResCNN). All models were trained using identical protocols: 5-fold cross-validation, 150 epochs, AdamW optimizer with cosine annealing, and fixed random seed (42) to have a fair comparison [33,34,35]. The comprehensive performance comparison is presented in Section 3.1.

### 2.5. Training Procedure

To train and evaluate our models, we employed a five-fold cross-validation scheme on the 2349 trials. For each fold, the data were partitioned into a training set (80.0%) and a test set (20.0%), with 10% of the training set being used for the validation. We optimized network parameters using the AdamW optimizer (PyTorch version 2.9.0; initial learning rate $5\times 10^{-4}$, weight decay $1\times 10^{-4}$) [36]. All GPU computations were performed on NVIDIA A100-SXM4-80GB (NVIDIA Corporation, Santa Clara, CA, USA) with CUDA (version 12.6). The learning rate schedule was varied depending on the model being evaluated: for ResCNN and AttentionCNN, the learning rate followed a Cosine Warm Restarts schedule ($T_{0}=10$ epochs, $T_{\mathrm{mult}}=2$). For the ensemble evaluation, the models within each fold were trained using a One-Cycle policy [37]. Early stopping was applied in each run, with a patience of 15–20 epochs and a minimum required improvement (Δ) of 0.001 in the validation loss. The model checkpoint with the best validation loss was then used for the final testing.

### 2.6. Ensemble Strategy

To combine the complementary outputs of AttentionCNN and ResCNN without additional training, we employed a late fusion of their softmax probabilities. For a given trial *x*, let

(4) $$\begin{array}{cc} p_{\mathrm{Res}}\left(x\right) & =\mathrm{softmax}\left(m_{\mathrm{Res}}\left(x\right)\right), \end{array}$$

(5) $$\begin{array}{cc} p_{\mathrm{Att}}\left(x\right) & =\mathrm{softmax}\left(m_{\mathrm{Att}}\left(x\right)\right). \end{array}$$

We compute the ensemble probability as the arithmetic mean,

(6) $$p_{\mathrm{ens}}\left(x\right)=\frac{p_{\mathrm{Res}}\left(x\right)+p_{\mathrm{Att}}\left(x\right)}{2},$$

and assign an “odor” label if $p_{\mathrm{ens}}\left(x\right)>0.5$. This simple fusion does not require additional parameters and incurs minimal inference overhead. Under five-fold cross-validation, this probability averaging was performed within each fold: For each of the five splits, an AttentionCNN and a ResCNN were trained on the training portion, and their ensembled predictions were evaluated on the held-out test portion. The final reported metrics are the mean and standard deviation of the performance across these five folds.

### 2.7. Evaluation Metrics

We evaluated the classification performance using the following metrics:

(7) $$\begin{array}{cc} \mathrm{Accuracy} & =\frac{\mathrm{TP}+\mathrm{TN}}{\mathrm{TP}+\mathrm{TN}+\mathrm{FP}+\mathrm{FN}}, \end{array}$$

(8) $$\begin{array}{cc} \mathrm{Precision} & =\frac{\mathrm{TP}}{\mathrm{TP}+\mathrm{FP}}, \end{array}$$

(9) $$\begin{array}{cc} \mathrm{Recall}\;\left(\mathrm{Sensitivity}\right) & =\frac{\mathrm{TP}}{\mathrm{TP}+\mathrm{FN}}, \end{array}$$

(10) $$\begin{array}{cc} \mathrm{Specificity} & =\frac{\mathrm{TN}}{\mathrm{TN}+\mathrm{FP}}, \end{array}$$

(11) $$\begin{array}{cc} F1 & =2\times \frac{\mathrm{Precision}\times \mathrm{Recall}}{\mathrm{Precision}+\mathrm{Recall}}, \end{array}$$

where TP, TN, FP, and FN denote true positives, true negatives, false positives, and false negatives, respectively. Additionally, we computed the Area Under the Receiver Operating Characteristic Curve (AUC) and used confusion matrices to clarify detailed error distributions. All metrics were calculated within each fold of a five-fold cross-validation and reported as the mean ± standard deviation. Furthermore, to verify the reliability of the probability estimates, we compared the model output with the actual labels for each test trial to assess the calibration (for example, a predicted odor probability ∼80.0% typically indicated actual odor trials ∼80.0%).

## 3. Results

### 3.1. Architecture Comparison and Selection

Table 1 presents the systematic comparison of eight CNN architectures, justifying our selection of AttentionCNN and ResCNN for the ensemble model. The baseline Vanilla CNN (three convolutional layers) achieved 83.9% accuracy, establishing our performance floor. Simply adding depth without architectural innovations proved ineffective—the Deep CNN (six layers without skip connections) matched this performance at 83.9%, likely due to gradient degradation. Dilated convolutions (Dilated CNN: 83.0%) and increased channel width (Wide CNN: 82.1%) similarly failed to improve upon the baseline, while the Shallow CNN (79.9%) confirmed that insufficient model capacity limits the performance.

In contrast, our proposed architectures demonstrated clear advantages. ResCNN with squeeze-and-excitation residual blocks reached 85.6%, demonstrating the value of skip connections and channel attention for multi-electrode LFP classification. AttentionCNN with CBAM attention achieved 84.7%, learning complementary representations through its multi-scale convolutional branches. Combining both architectures via ensemble averaging yielded the highest performance (86.2% accuracy, 0.942 AUC), a 2.3 percentage point improvement over the baseline, validating our design choices.

### 3.2. Performance Metrics Distribution Across Models

Table 2 summarizes the comprehensive evaluation metrics for our three primary models across five-fold cross-validation. AttentionCNN achieved 84.7% accuracy with high specificity (92.2%), while ResCNN reached 85.6% accuracy with more balanced sensitivity (87.0%) and specificity (86.0%). The ensemble combined these complementary strengths, achieving 86.2% accuracy with 84.0% sensitivity and 90.0% specificity. These results indicate that (i) both architectural innovations yield strong single-trial detection, and (ii) ensemble fusion maintains peak accuracy while balancing the sensitivity and specificity for robust odor detection.

### 3.3. Learned Feature Representation

**Figure 2:**
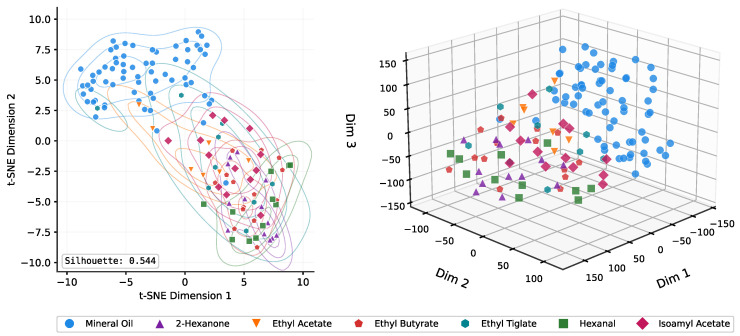
t-SNE visualization of ensemble feature embeddings colored by odor identity. Left: 2D projection with density contours (silhouette score = 0.544). Right: 3D projection showing spatial relationships between odor clusters. Mineral oil (control) forms a distinct cluster separated from the various odorants, with some overlap in the boundary region corresponding to harder-to-classify trials.

Beyond the quantitative metrics, we validated that our model learned biologically significant features. Figure 2 visualizes the feature space learned by the network using t-SNE [38]. The per-odor colored embedding (Figure 2) achieves a silhouette score of 0.544. Mineral oil trials cluster distinctly from odor trials, while the relative positions of individual odorants correlate with their classification accuracy—hexanal and 2-hexanone form tight clusters far from the control region, whereas ethyl acetate trials appear closer to the boundary, consistent with its lower detectability (detailed in Section 3.4). The 3D projections provide additional perspective, clearly showing the spatial separation between the control and odor-evoked neural patterns.

The partial overlap seen in the t-SNE embedding reflects differences in how well the neural responses to different odorants can be distinguished from the baseline. Our per-odor analysis (Table 3) shows that odorants producing weaker neural signatures, such as ethyl acetate (67.1% accuracy), account for most of the overlap region. In contrast, odorants like hexanal (96.3% accuracy) form tight well-separated clusters. This pattern is consistent with what we know about glomerular activation [39], where some odorant molecules produce more distinct responses than others.

### 3.4. Per-Odor Classification Performance

**Figure 3:**
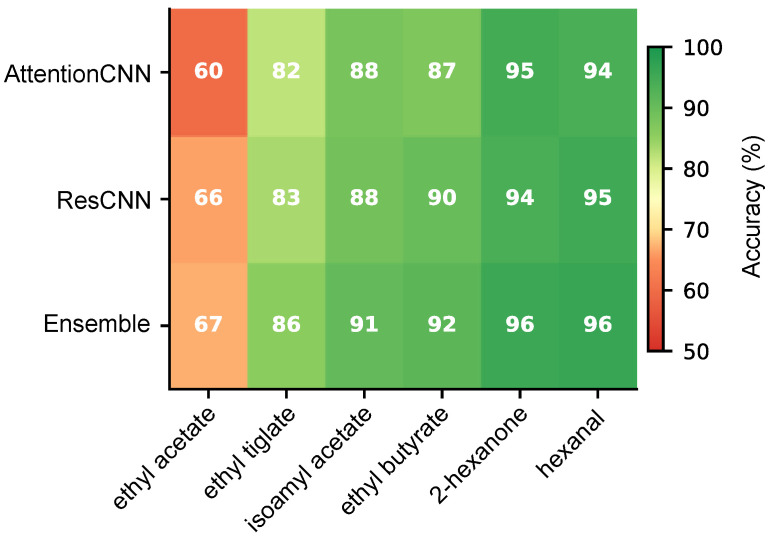
Per-odor classification accuracy for binary detection (odor vs. mineral oil) shown as a heatmap. Numbers indicate mean accuracy across five-fold cross-validation. Aldehydes and ketones show higher detectability compared to esters.

When we trained separate classifiers for each odorant against the mineral oil control, we found substantial differences in how well each could be detected (Table 3, Figure 3). Hexanal and 2-hexanone both achieved over 95% accuracy, suggesting these compounds produce particularly distinctive neural responses [40]. Ethyl acetate, on the other hand, proved much harder to detect at only 67.1% accuracy. This 29.2 percentage point range tells us that the olfactory bulb does not encode all odors with equal clarity. This pattern indicates that aldehydes (hexanal) and ketones (2-hexanone) show higher detectability compared to esters (ethyl acetate, ethyl tiglate), likely due to their distinct molecular properties. Factors like receptor binding characteristics and the spatial pattern of glomerular activation [41,42] determine how discriminable each odor’s neural signature is from the baseline.

### 3.5. Concentration-Dependent Detection

We tested whether our detection framework has a concentration threshold by analyzing ethyl butyrate across four concentration levels spanning two orders of magnitude (Table 4). At 0.03% *v*/*v*, the classification was no better than a binary random chance (51.3%), indicating the neural response at this concentration is too weak to detect [43,44]. The performance rose to 60.8% at 0.1% *v*/*v* and reached 75.2% at 0.3% *v*/*v*, which we take as the practical detection threshold. At the highest concentration tested (1.0% *v*/*v*), the accuracy reached 86.4%. This monotonic increase in accuracy with concentration mirrors the dose–response characteristics of sensory neurons [45,46], suggesting our classifier is tracking the strength of the neural response rather than picking up on artifacts.

### 3.6. Inference Speed Analysis

To assess the real-time capability, we benchmarked the inference speed across a CPU and three GPU platforms (Table 5). The lightweight ensemble architecture (461.4 K parameters) achieves single-sample inference in just 2.58 ms on a CPU—faster than the GPU, due to the kernel launch overhead at small batch sizes. Given the 1000 ms trial duration, this corresponds to a real-time factor of 388×, enabling deployment on standard hardware without GPU acceleration. For batch processing of large datasets, GPU throughput reaches 11,780 samples/second at batch size 64, facilitating rapid offline analysis.

### 3.7. Ensemble Prediction Confidence

**Figure 4:**
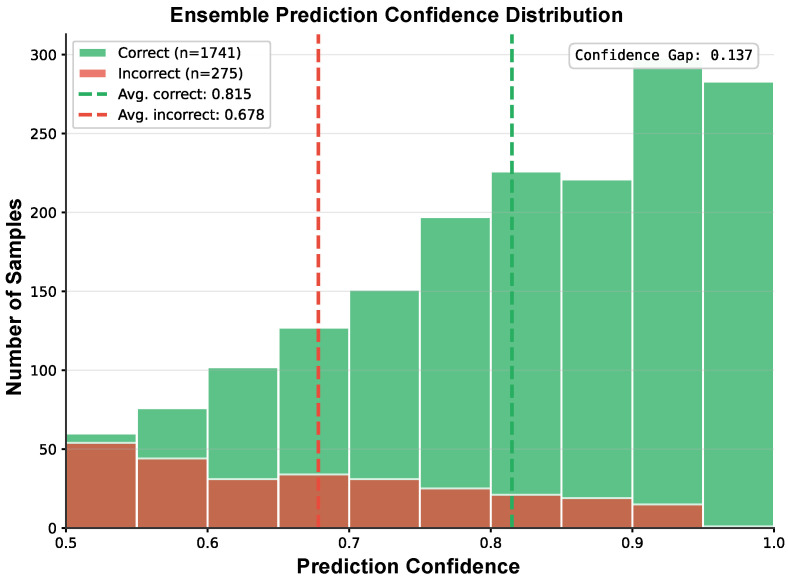
Histogram of ensemble prediction confidence for individual test trials, with correct predictions shown in green and incorrect predictions in red.

Figure 4 displays the distribution of the ensemble prediction confidence for correct and incorrect classifications. The horizontal axis shows the confidence level, and the vertical axis indicates the frequency of trials within each confidence bin. The green bars correspond to correct predictions with higher confidence values (mean confidence ≈ 0.815), while the red bars (incorrect predictions) are distributed more broadly with lower mean confidence (≈0.678). This suggests that the ensemble classifier is well calibrated, as it expresses higher confidence on correct predictions and lower confidence on misclassifications.

## 4. Discussion

### 4.1. Overcoming Prior Limitations

The findings presented in this study offer two primary contributions to the field of olfactory decoding. The first contribution is related to methodological advance: we establish the feasibility of accurate single-trial odor detection using deep learning on spectral features. This validates our first hypothesis that these signals contain sufficient information for robust classification without averaging. The second contribution is related to neurological insight: we demonstrate that this performance can be achieved using signals from the olfactory bulb alone. This supports our second hypothesis that the initial stages of olfactory processing are sufficient for the fundamental task of presence detection for seven odors, without requiring contributions from higher cortical regions.

Prior non-invasive work on human olfactory registration has shown lower performance. Rajabi et al. evaluated logistic regression and an end-to-end 1D ResNet on EEG and EBG signals, finding that their linear baseline remained at AUC ≈ 50%, and their ResNet-1D could push AUC into the high-50% range (e.g., 56.6% for scalp-EBG, 58.0% for EEG) [23]. The difficulty of cross-subject generalization with EEG is further shown by the work of Ezzatdoost et al., who achieved 64.3% accuracy on a more complex four-odor identification task using handcrafted nonlinear features [19]. Similarly, Kato et al. demonstrated that, while odor representations in human EEG can be decoded within 100 ms post-stimulus onset, achieving reliable classification remains challenging due to signal quality limitations [47]. This demonstrates that current non-invasive recordings lack sufficient SNR for accurate single-trial odor detection [18,48] and highlights the advantage of LFP signals.

Table 6 places our results within the larger context of neural odor decoding. Although direct quantitative comparison is limited by differences in recording modality or experimental procedure, the substantial performance gap corroborates our methodological assumption that high SNR neural recordings are a prerequisite for robust single-trial odor detection. The 22–29 percentage point accuracy gain observed is consistent with the recognized difference in signal fidelity between intracranial LFP recordings versus non-invasive scalp modalities such as EEG or EBG [13], definitively validating our methodological assumptions. Finally, we acknowledge that the performance differential may be influenced by interspecific differences in olfactory processing between mice and humans.

### 4.2. Methodological Contributions

This work establishes a reliable neural-based binary odor detection system, providing both methodological advances and biological insights. Our ensemble approach effectively combines complementary CNN architectures to achieve reliable performance. The clear separation observed in our t-SNE visualization (Figure 2) and the high confidence levels for correct predictions (Figure 4) further validate that our models have learned biologically meaningful features that capture fundamental differences between odor-presence and odor-absence neural states. Compared to prior EEG-based studies, our LFP approach offers higher spatial resolution and direct access to OB circuitry, enabling the extraction of rich spectro-temporal signatures that were previously inaccessible.

### 4.3. Limitations and Future Directions

Our concentration analysis (Section 3.5) shows that the detection performance depends on the stimulus strength, with a practical threshold at 0.3% *v*/*v* for ethyl butyrate. Below this concentration, neural responses become too weak for reliable single-trial classification. This finding has important implications for real-world applications, where target analyses may be present at trace concentrations. Future work should systematically characterize detection thresholds across all six odorants and investigate whether multi-trial averaging or more sensitive pre-processing methods can lower these limits.

Our evaluation focuses on monomolecular odorants at a fixed concentration (0.3% *v*/*v*) in head-fixed mice, leaving important questions about generalization to complex mixtures [49], varying concentrations, and naturalistic behavioral conditions. The invasive nature of LFP recordings also limits immediate translational applications, though our findings provide crucial validation of neural-based odor detection principles. Future work will systematically extend our approach to diverse odor mixtures and concentration ranges, validate its performance in freely moving animals, and investigate non-invasive recording modalities to assess translational feasibility.

## 5. Conclusions

This study demonstrates that deep neural networks can achieve robust single-trial odor detection from olfactory bulb LFPs, establishing two key findings. First, spectral features within single-trial LFPs provide sufficient information for accurate odor presence classification, achieving 86.2% accuracy and 0.942 AUC—substantially outperforming prior non-invasive approaches. Second, signals from the olfactory bulb alone are adequate for this task, without requiring downstream cortical processing. Our ensemble of AttentionCNN and ResCNN architectures leverages complementary feature extraction strategies to capture biologically significant neural signatures, as confirmed by t-SNE visualization and prediction confidence analysis. These findings establish the feasibility of LFP-based odor sensing and provide a foundation for future development of neural-based detection systems.

## Figures and Tables

**Table 1:** Performance comparison of CNN architectures for binary odor detection. All models are trained with identical protocols (five-fold CV, 150 epochs, AdamW optimizer, seed = 42).

Architecture	Accuracy (%)	F1 (%)	AUC
**Ensemble**	**86.2 ± 2.8**	**85.3 ± 3.4**	**0.942 ± 0.011**
**ResCNN**	**85.6 ± 2.8**	**85.0 ± 3.5**	**0.931 ± 0.021**
**AttentionCNN**	**84.7 ± 2.2**	**83.6 ± 2.4**	**0.921 ± 0.016**
Vanilla CNN	83.9 ± 2.4	83.2 ± 3.0	0.911 ± 0.021
Deep CNN	83.9 ± 1.4	83.3 ± 3.0	0.922 ± 0.016
Dilated CNN	83.0 ± 4.1	83.1 ± 4.6	0.912 ± 0.034
Wide CNN	82.1 ± 1.8	81.1 ± 2.9	0.906 ± 0.022
Shallow CNN	79.9 ± 2.7	77.4 ± 3.7	0.875 ± 0.015

Bold entries indicate the three primary architectures proposed in this study.

**Table 2:** Comprehensive performance metrics for AttentionCNN, ResCNN, and ensemble models (mean ± SD over five folds). Abbreviations: Acc. = accuracy; F1 = F1 score; AUC = area under the receiver operating characteristic curve; Sens. = sensitivity; Spec. = specificity.

Model	Acc. (%)	F1 (%)	AUC	Sens. (%)	Spec. (%)
AttentionCNN	84.7 ± 2.2	83.6 ± 2.4	0.921 ± 0.016	76.0 ± 2.5	92.2 ± 1.8
ResCNN	85.6 ± 2.8	85.0 ± 3.5	0.931 ± 0.021	87.0 ± 1.9	86.0 ± 2.2
Ensemble	86.2 ± 2.8	85.3 ± 3.4	0.942 ± 0.011	84.0 ± 2.0	90.0 ± 1.7

**Table 3:** Per-odor classification performance using the ensemble model. Each row shows binary classification (specific odorant vs. mineral oil) using the same five-fold cross-validation protocol. All odorants tested at 0.3% *v*/*v* concentration.

Odorant	Trials	Accuracy (%)	Interpretation
Hexanal	337	96.3 ± 1.5	Excellent separability
2-Hexanone	335	95.6 ± 2.6	Excellent separability
Ethyl butyrate	335	91.5 ± 2.7	High separability
Isoamyl acetate	336	90.6 ± 2.8	High separability
Ethyl tiglate	335	85.5 ± 2.4	Moderate separability
Ethyl acetate	335	67.1 ± 5.0	Low separability

**Table 4:** Concentration-dependent classification performance for ethyl butyrate. Statistical significance tested against chance level (50%) using one-sample *t*-test. The detection threshold is defined as the lowest concentration achieving both p<0.001 and accuracy >70%.

Concentration (% *v*/*v*)	Accuracy (%)	vs. Chance	Detection Status
0.03	51.3 ± 5.0	p>0.05	At chance level
0.10	60.8 ± 5.6	p<0.05	Marginal detection
0.30	75.2 ± 2.7	p<0.001	Reliable detection
1.00	86.4 ± 3.4	p<0.001	Robust detection

**Table 5:** Inference speed benchmarking across different hardware platforms. Latency represents single-sample (batch size = 1) processing time. All measurements are mean ± standard deviation over 200 runs after 50 warm-up iterations. Model sizes: AttentionCNN (155.1 K params, 0.6 MB), ResCNN (306.3 K params, 1.2 MB), ensemble (461.4 K params, 1.8 MB).

Model	Hardware	Latency (ms)	Throughput (Samples/s)	Memory (MB)
AttentionCNN	CPU	1.03 ± 0.05	970	–
	Tesla T4	1.86 ± 0.10	539	19
	NVIDIA L4	1.77 ± 0.02	564	19
	A100-80GB	1.76 ± 0.04	569	19
ResCNN	CPU	1.75 ± 0.01	571	–
	Tesla T4	3.86 ± 0.05	259	16
	NVIDIA L4	4.18 ± 0.15	239	16
	A100-80GB	3.86 ± 0.01	259	16
Ensemble	CPU	2.58 ± 0.01	387	–
	Tesla T4	5.52 ± 0.04	181	22
	NVIDIA L4	5.89 ± 0.06	170	22
	A100-80GB	5.56 ± 0.07	180	22

**Table 6:** Performance comparison across recording modalities and species. Note: Direct comparison is limited due to differences in species (human vs. mouse), recording invasiveness (scalp vs. intracranial), and experimental protocols.

Study	Species	Signal Type	Accuracy (%)	Task
Rajabi et al. [23]	Human	EEG (non-inv.)	58.0	Odor vs. Blank
Rajabi et al. [23]	Human	EBG (non-inv.)	57.0	Odor vs. Blank
Ezzatdoost et al. [19]	Human	EEG (non-inv.)	64.3	4-Odor Identity
**Our Work**	**Mouse**	**OB-LFP (inv.)**	**86.2**	**Odor vs. Blank**

Bold entry indicates the results from the present study.

## Data Availability

The data used in this study are publicly available at CRCNS.org (https://doi.org/10.6080/K00C4SZB).

## References

[1] Sanislav T., Mois G.D., Zeadally S., Folea S., Radoni T.C., Al-Suhaimi E.A. (2025). A Comprehensive Review on Sensor-Based Electronic Nose for Food Quality and Safety. Sensors.

[2] Kim C., Lee K.K., Kang M.S., Shin D.M., Oh J.W., Lee C.S., Han D.W. (2022). Artificial olfactory sensor technology that mimics the olfactory mechanism: A comprehensive review. Biomater. Res..

[3] Deng H., Chen Z., Feng P., Tian L., Zong H., Nakamoto T. (2025). Recent Advances and Applications of Odor Biosensors. Electronics.

[4] Dennler N., Drix D., Warner T.P.A., Rastogi S., Della Casa C., Ackels T., Schaefer A.T., van Schaik A., Schmuker M. (2024). High-speed odor sensing using miniaturized electronic nose. Sci. Adv..

[5] Kim T., Kim Y., Cho W., Kwak J.-H., Cho J., Pyeon Y., Kim J.J., Shin H. (2024). Ultralow-power single-sensor-based e-nose system powered by duty cycling and deep learning for real-time gas identification. ACS Sens..

[6] Shor E., Herrero-Vidal P., Dewan A., Uguz I., Curto V.F., Malliaras G.G., Savin C., Bozza T., Rinberg D. (2022). Sensitive and robust chemical detection using an olfactory brain-computer interface. Biosens. Bioelectron..

[7] Lu Q., Yi M., Jiang J. (2025). Bioelectronic nose for ultratrace odor detection via brain-computer interface with olfactory bulb electrode arrays. Biosens. Bioelectron..

[8] Qin C., Wang Y., Hu J., Wang T., Liu D., Dong J., Lu Y. (2023). Artificial Olfactory Biohybrid System: An Evolving Sense of Smell. Adv. Sci..

[9] Morozova M., Bikbavova A., Bulanov V., Lebedev M.A. (2023). An olfactory-based brain-computer interface: Electroencephalography changes during odor perception and discrimination. Front. Behav. Neurosci..

[10] Ninenko I., Medvedeva A., Efimova V.L., Kleeva D.F., Morozova M., Lebedev M.A. (2024). Olfactory neurofeedback: Current state and possibilities for further development. Front. Hum. Neurosci..

[11] Zhu P., Liu S., Tian Y., Chen Y., Chen W., Wang P., Du L., Wu C. (2022). In vivo bioelectronic nose based on a bioengineered rat realizes the detection and classification of multiodorants. ACS Chem. Neurosci..

[12] Einevoll G.T., Kayser C., Logothetis N.K., Panzeri S. (2013). Modelling and analysis of local field potentials for studying the function of cortical circuits. Nat. Rev. Neurosci..

[13] Buzsáki G., Anastassiou C.A., Koch C. (2012). The origin of extracellular fields and currents—EEG, ECoG, LFP and spikes. Nat. Rev. Neurosci..

[14] Pesaran B., Vinck M., Einevoll G.T., Sirota A., Fries P., Siegel M., Truccolo W., Schroeder C.E., Srinivasan R. (2018). Investigating large-scale brain dynamics using field potential recordings: Analysis and interpretation. Nat. Neurosci..

[15] Yang Q., Zhou G., Noto T., Templer J.W., Schuele S.U., Rosenow J.M., Lane G., Zelano C. (2022). Smell-induced gamma oscillations in human olfactory cortex are required for accurate perception of odor identity. PLoS Biol..

[16] Arpaia P., Cataldo A., Criscuolo S., De Benedetto E., Masciullo A., Schiavoni R. (2022). Assessment and Scientific Progresses in the Analysis of Olfactory Evoked Potentials. Bioengineering.

[17] Ninenko I., Kleeva D.F., Bukreev N., Lebedev M.A. (2023). An experimental paradigm for studying EEG correlates of olfactory discrimination. Front. Hum. Neurosci..

[18] Iravani B., Arshamian A., Ohla K., Wilson D.A., Lundström J.N. (2020). Non-invasive recording from the human olfactory bulb. Nat. Commun..

[19] Ezzatdoost K., Hojjati H., Aghajan H. (2020). Decoding olfactory stimuli in EEG data using nonlinear features: A pilot study. J. Neurosci. Methods.

[20] Abbasi N.I., Bose R., Bezerianos A., Thakor N.V., Dragomir A. (2019). EEG-based classification of olfactory response to pleasant stimuli. Proceedings of the 41st Annual International Conference of the IEEE Engineering in Medicine and Biology Society (EMBC).

[21] Hou H.R., Han R.X., Zhang X.N., Meng Q.H. (2022). Pleasantness Recognition Induced by Different Odor Concentrations Using Olfactory Electroencephalogram Signals. Sensors.

[22] Huart C., Legrain V., Hummel T., Rombaux P., Mouraux A. (2012). Time-Frequency Analysis of Chemosensory Event-Related Potentials to Characterize the Cortical Representation of Odors in Humans. PLoS ONE.

[23] Rajabi N., Zanettin I., Ribeiro A.H., Vasco M., Björkman M., Lundström J.N., Kragic D. (2025). Exploring the feasibility of olfactory brain-computer interfaces. Sci. Rep..

[24] Livezey J.A., Glaser J.I. (2021). Deep learning approaches for neural decoding across architectures and recording modalities. Brief. Bioinform..

[25] Hossain K.M., Islam M.A., Hossain S., Nijholt A., Ahad M.A.R. (2022). Status of deep learning for EEG-based brain–computer interface applications. Front. Comput. Neurosci..

[26] Bolding K.A., Franks K.M. (2018). Recurrent cortical circuits implement concentration-invariant odor coding. Science.

[27] Bolding K.A., Franks K.M. (2018). Simultaneous Extracellular Recordings from the Mouse Olfactory Bulb and Piriform Cortex in Response to Odor Stimuli. CRCNS.org.

[28] Lepousez G., Lledo P.M. (2013). Odor Discrimination Requires Proper Olfactory Fast Oscillations in Awake Mice. Neuron.

[29] Kay L.M. (2003). Two Species of Gamma Oscillations in the Olfactory Bulb: Dependence on Behavioral State and Synaptic Interactions. J. Integr. Neurosci..

[30] Welch P.D. (1967). The use of fast Fourier transform for the estimation of power spectra: A method based on time averaging over short, modified periodograms. IEEE Trans. Audio Electroacoust..

[31] Rousseeuw P.J., Croux C. (1993). Alternatives to the Median Absolute Deviation. J. Am. Stat. Assoc..

[32] Hu J., Shen L., Sun G. (2018). Squeeze-and-Excitation Networks. Proceedings of the IEEE/CVF Conference on Computer Vision and Pattern Recognition (CVPR).

[33] Lawhern V.J., Solon A.J., Waytowich N.R., Gordon S.M., Hung C.P., Lance B.J. (2018). EEGNet: A compact convolutional neural network for EEG-based brain-computer interfaces. J. Neural Eng..

[34] Schirrmeister R.T., Springenberg J.T., Fiederer L.D.J., Glasstetter M., Eggensperger K., Tangermann M., Hutter F., Burgard W., Ball T. (2017). Deep learning with convolutional neural networks for EEG decoding and visualization. Hum. Brain Mapp..

[35] Walther D., Viehweg J., Haueisen J., Mäder P. (2023). A systematic comparison of deep learning methods for EEG time series analysis. Front. Neuroinform..

[36] Kingma D.P., Ba J. Adam: A Method for Stochastic Optimization. Proceedings of the 3rd International Conference on Learning Representations (ICLR).

[37] Smith L.N. (2018). A disciplined approach to neural network hyper-parameters: Part 1—learning rate, batch size, momentum, and weight decay. arXiv.

[38] van der Maaten L., Hinton G. (2008). Visualizing Data using t-SNE. J. Mach. Learn. Res..

[39] Burton S.D., Brown A., Eiting T.P., Youngstrom I.A., Rust T.C., Schmuker M., Wachowiak M. (2022). Mapping odorant sensitivities reveals a sparse but structured representation of olfactory chemical space by sensory input to the mouse olfactory bulb. eLife.

[40] Shani-Narkiss H., Beniaguev D., Segev I., Mizrahi A. (2023). Stability and flexibility of odor representations in the mouse olfactory bulb. Front. Neural Circuits.

[41] Bhattacharjee A.S., Konakamchi S., Turaev D., Vincis R., Nunes D., Dingankar A.A., Spors H., Carleton A., Kuner T., Abraham N.M. (2019). Similarity and strength of glomerular odor representations define a neural metric of sniff-invariant discrimination time. Cell Rep..

[42] Economo M.N., Hansen K.R., Wachowiak M. (2016). Control of mitral/tufted cell output by selective inhibition among olfactory bulb glomeruli. Neuron.

[43] Parabucki A., Bizer A., Morris G., Munoz A.E., Bala A.D.S., Smear M., Shusterman R. (2019). Odor concentration change coding in the olfactory bulb. eNeuro.

[44] Bolding K.A., Franks K.M. (2017). Complementary codes for odor identity and intensity in olfactory cortex. eLife.

[45] Rolls E.T., Baker K.L., Bhattacharjee A.S., Verhagen J.V. (2023). Odor encoding by signals in the olfactory bulb. J. Neurophysiol..

[46] Shusterman R., Sirotin Y.B., Smear M.C., Ahmadian Y., Rinberg D. (2018). Sniff invariant odor coding. eNeuro.

[47] Kato M., Okumura T., Tsubo Y., Honda J., Sugiyama M., Touhara K., Okamoto M. (2022). Spatiotemporal dynamics of odor representations in the human brain revealed by EEG decoding. Proc. Natl. Acad. Sci. USA.

[48] Iravani B., Schaefer M., Wilson D.A., Arshamian A., Lundström J.N. (2021). The human olfactory bulb processes odor valence representation and cues motor avoidance behavior. Proc. Natl. Acad. Sci. USA.

[49] Zak J.D., Reddy G., Vergassola M., Murthy V.N. (2024). Distinct information conveyed to the olfactory bulb by feedforward input from the nose and feedback from the cortex. Nat. Commun..

